# Tennessee State Medical Society

**Published:** 1888-05

**Authors:** 


### TENNESSEE STATE MEDICAL SOCIETY.

*Editors Atlanta Medical and Surgical Journal:*

The Tennessee State Medical Society held its fifty-fifth annual meeting in Knoxville, April 10th, 11th and 12th. The following is a list of papers presented:

Laparotomy Practically Considered—Richard Douglas, Nashvillle.

The Germ Theory of Disease—H. P. Coyle, Knoxville.

Fissures and Ulcers of the Rectum—T. J. Happel, Trenton.

Diphtheria and its Treatment—Jno. M. Boyd, Knoxville.

Philosophy of Fever—C. W. Beaumont, Clarkesville.

Infant Feeding—G. A. Baxter, Chattanooga.

Syphilis and Marriage—W. F. Glenn, Nashville.

Intestinal Obstruction—T. L. Maddin.

Some Suggestions in Regard to the Treatment of the Insane—C. C. Fite, Knoxville.

Resection of the First Metatarsal Bone of the Foot for the Cure of Bunion—S. T. Armstrong, New York.

Some Observations and Thoughts on General Paralysis—M. Campbell, Knoxville.

Cholera in Tennessee from 1833 to 1835—J. B. Lindsley, Nashville.

Points in the Differentiation of Serous Iritis from Sub-acute Glaucoma—N. C. Steele, Chattanooga.

Materia Medica and New Remedies--C. N. Cooper, Cleveland.

Menorrhagia and Metrorhagia—C. E. Ristine, Knoxville.

The Eye and Its Abuse—Frank Trester Smith, Chattanooga.

Dr Douglas reported four cases and gave the results of his experience. Dr. Coyle's paper was well received and elicited considerable discussion. Dr. Boyd gave his experience in treating diphtheria with large doses of tr. veratrum viride (teaspoonful of Norwood's tincture). With it he had saved cases that otherwise would certainly have died according to the old methods. He was given a vote of thanks by the society, and it was the general consensus of opinion that the method should be tried and results reported at the next meeting. Dr. Glenn's paper on syphilis elicited considerable discussion, as did also Dr. Baxter's.

Dr. T. L. Maddin advocated the treatment of intussusception by means of morphine and atropine, believing it to be due to spasm of the bowel. He reported four cases, one of which occurred in his own person.

Dr. Trester Smith introduced a new set of test type corresponding to No. xx. of Snellen. The patient is placed at twenty feet and advances until he can read the type, when the distance is taken for the numerator and twenty for the denominator of the fraction, which will indicate the visual power. The advantage of the test lies in its simplicity.

Early in the session the society voted against attending banquets or engaging in anything that would interfere with the scientific work for which they had come together. A reception was given, however, by the Chilhowie Club on the night after the second day's session.

The President's address on Materialism in Medicine was delivered in the Opera House to a good audience.

The business of the society was completed Thursday morning. In the afternoon the public institutions were visited in carriages provided through the courtesy of the Committee of Arrangements. Yours very truly,

S.

*Chattanooga, Tenn., April 18th, 1888.*

### Editorial.

### ITEMS.

Mr. Logan, of R. A. Robinson & Co., Louisville, Ky., made many new friends for the specialties of his house.

Lea Brothers & Co., the publishers, of Philadelphia, were represented at Montgomery and Rome by good agents.

I. Phillips, Atlanta's instrument man, was at both Montgomery and Rome, and as usual sold a large amount of goods.

Mr. Carr, Fairchild's digestive ferments, and Mr. Smith, Carnrick's soluble food, two clever gentlemen, were at Rome.

Mr. T. Engelbach, surgical instruments, New Orleans, and John Wyeth & Bro., of Philadelphia, had exhibits at Montgomery.

The exhibits of medical and surgical supplies, both at Montgomery and Rome, were unusually fine and the Doctors were freely sampled.

Dr. J. T. Galt, of Norfolk, Virginia, a genial, wide-awake practitioner, represented the Old Dominion at the meeting of the Association in Rome.

The Journal's thanks are due for many kind words. We are trying to make this a good journal, and are glad to know that you think we are successful.

Mr. Frost, of The Mellier Drug Co., St. Louis, Mo., was at Rome with a good display of drugs and instruments and made many friends both for himself and house.

Eli Lilly & Co., of Indianapolis, Ind., had as handsome a display as we ever saw. It is a good house, and we learn they are establishing a good connection in the South.

DR. A. VANDER VEER, of Albany, New York, passed through the city recently on his return from a pleasure trip to Florida. He is doubtless too well known to our readers to need any statement of his success as a surgeon, a gynæcologist and a writer. As Professor of Surgery in the Albany Medical College, and as the author of an article in the American System of Gynæcology, besides many other articles scattered through the medical journals, he has acquired a reputation which is national in its extent.

AMONG the visitors to Rome at the recent meeting of the Medical Association of Georgia, we had the pleasure of meeting Dr. George H. Rohé and Dr. J. H. Branham, of Baltimore. These gentlemen were on a brief pleasure trip to the South, and took in the meeting, much to the gratification of all who had the opportunity of forming their acquaintance. Dr. Rohé is Professor of Hygiene and Clinical Dermatology, and Dr. Branham, Demonstrator of Anatomy in the College of Physicians and Surgeons. Both are surgeons to Bayview Hospital. If the Baltimore physicians can be judged by these two samples, we earnestly hope that we may meet many more of them in the future.

DR. K. P. MOORE, of Macon, Georgia, was elected Secretary of the Medical Association of Georgia at the meeting recently held in Rome. The Doctor is one of the most popular and useful members of the body, enjoying the highest confidence of the community in which he lives, and the greatest respect of his professional brethren. From merit alone he has occupied every position of trust in the gift of the Association, from the Presidency down, with an ability that has crowned all his labors with success. He accepted the Secretary's office under protest, but with a devotion and loyalty to the cause yielded his individual preferences for the good of the Association.

DR. J. A. FITZ HUGH, a former practitioner of Amesbury, Massachusetts, has located in Atlanta, and will devote his attention to the specialty of Dermatology. He has just returned from London, where he has spent two years in the study of his specialty under the leading Dermatologist of that city. We believe that a wide field is open to him.

THE Medical Association of Georgia paid a high but merited compliment to Dr. J. S. Todd in electing him to the Presidency of that distinguished body. Dr. Todd is one of the most successful and deservedly popular of Georgia's physicians. He is a gentleman in all the high essentials of the term, and a physician of the noblest attributes of a noble profession. Our own Dr. J. B. S. Holmes received the compliment of the Vice-Presidency, and is worthy of even higher honors, which he will doubtless receive at the next convention. In the whole Association there is not a better and more successful practitioner than Dr. Holmes. The *Tribune* congratulates the Georgia doctors upon the selection of two men so fitted for responsible positions and so eminently worthy of honor conferred upon them.—*Rome Tribune.*

DEXTROCARDIA.—A case of right-sided position of the heart was recently shown to the Vienna K. K. Gesellschaft der Aerzte by Dr. A. Gruss. The patient, a young woman, aged 20, was badly developed, and complained much of dyspnœa and giddiness. There had been some improvement of late years. The pulsations of the heart on the right side of the sternum were noted at birth, and there had always been some cyanosis and feeling of coldness. There was no cardiac dullness on the left side; on the right it began at the fourth rib, extending inwards to the right sternal border, and downwards, being lost in the liver dullness. Auscultation revealed in the second left intercostal space a distinct systolic murmur, and there was a loud diastolic sound. The murmur was heard in both carotids. The pulse was very small, but otherwise normal. The abdominal organs were normally situated. The diagnosis made was pure dextrocardia and congenital pulmonary stenosis without malposition of the viscera in general. Von Bamberger concurred in the diagnosis, and remarked that Professor Schrötter had lately stated that no single case of pure dextrocardia had ever been proved; whereas, all anatomists of great experience—for example, Rokitansky, Friedberg, Forster, etc.—had mentioned such cases, and he himself had seen two. The murmur was due to congenital stenosis, for the acquired kind was extremely rare, though he and Dittritch had described such a case (due to a kick of a horse). The loud second sound excluded the aorta as its origin. He thought also that the great vessels were not transposed; the case had gone on too favorably for that supposition. Professor Kundrat said that the great vessels might be transposed, the position of the septum to some extent correcting the malposition.—*British Medical Journal.*

EARLY RISING AND LONGEVITY.—Professor Humphry's recent Collective Investigation Report on Aged Persons, published in the *Journal*, contains some very positive evidence on a matter which has already engaged the attention of moralists as well as physicians. "The opportunity for nutrition to do its restorative work was in nearly all provided by the faculty of 'good sleeping,' to which was commonly added its appropriate attendant, the habit of 'early rising.'" Thus there is a relation between early rising and longevity. No doubt many people will hastily seize upon the sentence just quoted and employ it in edifying lectures or essays for the perusal of youth, or embody in it popular medical works. Important qualifications follow in Dr. Humphry's report, but they are likely to be overlooked. Doubtless the habit of early rising is, in itself, healthy; most of all, it is a good sign of health when it evidently signifies rapid recovery from fatigue. Again, it usually denotes a strong will, the gift as a rule of a good physical constitution, or at least the safeguard of average bodily strength. Late risers are generally either invalids or persons of bad habits, idlers who are never free from other vices besides idleness. The nervous exhaustion which keeps a man wakeful throughout the small hours produces sleep late in the morning. This exhaustion is invariably due to one of several life-shortening influences, especially anxiety or indiscretion in diet or drink. Early rising is thus rather one effect of certain favorable influences, another result of which is longevity, than a cause of longevity. To turn a weakly man out of bed every morning at 7 o'clock will not prolong his life. It will be noted that by "good sleeping" Professor Humphry signifies quick sleeping; "that is, the reparative work which has to be done in sleep is done briskly and well." Here again we have an effect of a cause; but preventing a weakly subject from sleeping more than four or five hours nightly would not cause him to live long, but would rather tend to shorten his life. Equally important are Professor Humphry's observations which show that by "early" he does not entirely mean the time by the clock. The word "has a relative significance with reference to the time of going to bed. A person who retires to rest four hours after midnight and gets up at 10 a. m. may be strictly regarded as an early 'riser.'" Thus early rising is synonymous, in long life histories, with short sleeping, which means rapid recovery from fatigue, a sign of bodily strength. These scientific facts in nowise contradict the alleged value of early rising as a practice to be cultivated by all persons in good health. It is excellent as a moral discipline and eminently healthy as a matter of fact. Most persons will eat three meals daily. When a man gets up late those meals will probably follow each other at too short intervals to be wholesome. When he is an early riser it will probably be otherwise. He can enjoy a good breakfast, and by the time for his lunch or midday dinner he will have an honest appetite.—*British Medical Journal.*

AT a recent meeting of the Wiener Medizinisches Doctoren-Collegium, Professor Edward Lang, of Vienna, read a paper on the Co-existence of Syphilis and Cancer. But little attention had been hitherto directed to the fact that syphilis sometimes formed the predisposing soil for the development of cancer, or that both diseases might be combined with each other. Setting aside some observations of comparatively little value which had been made by ancient authors on this subject, it was especially Hutchinson and Langenbeck, who, before Professor Lang, had directed attention to the simultaneous occurrence of syphilis and carcinoma. The speaker had observed such a combination four times. The first case was that of a patient who had been admitted in 1883 under his care when he was at Innsbruck. Besides an old-standing iritis and other symptoms of syphilis, he was suffering from serpiginous and gummatous ulcerations on the nose, the cheek and the angles of the eye. Most of the ulcers healed under anti-syphilitic treatment, except one, which gradually changed its character and assumed the form of an "ulcus rodens"—that is, a flat carcinoma of the skin. The patient was transferred to the surgical clinic, where he underwent an operation, and microscopical examination proved the simultaneous existence of both syphilis and carcinoma. The second case was that of a patient, aged 46, who had previously been treated for recent syphilis by Professor Lang. There were syphilitic infiltrations of the floor of the mouth and under the tongue, and, moreover, various syphilitic infiltrations over the occipital bone and the body. All these lesions subsided under the influence of a common anti-syphilitic treatment, except the infiltrations of the floor of the mouth, which became transformed into cancer. The third case was that of a man, aged about 30, who presented a syphilitic ulcer on the under lip. The ulcer disappeared under anti-syphilitic treatment, but relapse and transformation into carcinoma occurred a year later. In all these cases the correctness of the diagnosis was proved by the anatomical examination. The fourth case was shown to the Society. The patient had extensive scars, ulcerations as well as infiltrations on the forehead, loss of the entire nose, loss of substance on the upper lip, ulceration and perforation of the hard palate, perforation of the soft palate and cicatricial retraction of the uvula. On admission of the patient into the clinic of Professor Lang, in the middle of January last, some of the ulcerations were covered with a white, tallowy mass, which led Professor Lang to suggest the presence of a combination of syphilis with carcinoma. The microscopical examination, however, revealed only the presence of pus corpuscles and products of decomposition. The infiltrations on the right side of the forehead later on slightly diminished, but from four to five weeks later a vegetation on the anterior end of the ulceration of the hard palate appeared, the character of which was incompatible with that of syphilitic ulceration. A part of this vegetation was excised, and the microscopical examination of it, which was made by Professor Weichselbaum, showed that it was "epithelial carcinoma."—*British Medical Journal.*

GUAIACOL IN PHTHISIS.—Professor Frankel, who has repeatedly advocated the use of creosote in the treatment of phthisis, now recommends guaiacol as the effective constituent of creosote. The good effects of the latter are, Frankel thinks after nine years' experience of its effects in phthisis, unmistakable in a strictly defined class of cases. If used promiscuously in this disease, its good effects are distinctly evident in 16 or 20 out of 400 or 500 cases, and are due not to destruction of the bacillus tuberculosis, but to its favorable influence on the digestion. It has long been known that creosote is a mixture of different substances, and last summer Professor Penzoldt remarked that guaiacol appeared to be its strict therapeutical constituent. Since then Dr. Sahli, of Berne, has reported on the clinical uses of guaiacol, and Professor Frankel has been using it since the beginning of the year in the following mixture, which suits admirably—guaiacol, 13.5; tinct. gent., 30; sp. vini rect., 250; vini xerici, q. s. ad colat. 1,000—a dessertspoonful in a wineglassful of water two or three times a day. This mixture is superior to gelatine capsules or tolu balsam. Sommerbrodt's praise of creosote is hardly justified by facts. Frankel remarks on this point that his own patients were nearly all hospital patients carefully investigated, only nine being private patients, whereas Sommerbrodt speaks of five thousand private patients, but without giving details. In due time creosote (or rather, for the future, guaiacol) will take its proper place.—*British Medical Journal.*

IMPURE ANTIPYRINE.—The extraordinary demand for antipyrine is very much in excess of the supply, and great pressure is put upon the manufacturers to increase the amount of the manufactured article in the market. The consequence has been that due care has not been shown in the purification of the drug, a certain proportion of benzine having been detected in samples submitted to analysis according to Dr. Dujardin-Beaumetz. This impurity may account for some of the toxic symptoms which have been reported, such as cutaneous eruptions, gastric troubles, and even grave cerebral symptoms, more particularly in the aged.—*British Medical Journal.*

THE LESSON OF LIFE.—There have been few physicians whose death has caused so wide-spread and sincere grief as has that of Dr. Agnew. This was due, we take it, largely not so much to his professional eminence as to the exceptional purity and elevation of his character. Dr. Agnew was a Christian gentleman in the best sense of the word; devout without bigotry, sincere and earnest without pretense or intolerance. He impressed those whom he met as singularly free from pettiness of spirit, jealousy or ignoble prejudices. Generous and liberal toward those with whom he disagreed, no personal attacks ever seemed to crown his anger or call out vindictiveness. He lived in a region quite above the smaller passions that consume so many.

His was the kind of spirit which we should be glad to see more widely diffused, not only in our profession, but everywhere. The liberality of mind, the wide and generous interest in everything that would help his fellow-man, were distinguishing traits in the character of the late Dr. Agnew. We can only hope that they will be remembered and imitated.—*Medical Record.*

A MEDICAL "TRUST."—A physician of Spencerville, O., writes to the *Medical Standard:* "The physicians of Spencerville and vicinity have combined for protection against delinquent debtors. The plan adopted is as follows: The physicians make out and exchange lists of patients who are delinquent debtors. All agree to refuse medical aid to such delinquents except for cash. The plan has worked like a charm, and many old debts are being paid. Let physicians elsewhere do likewise."

DRUGGISTS AND THE PRESCRIPTION OF POISONS.—The Indiana Legislature has passed a law declaring that "From and after the passage of this act, no pharmacist, druggist, apothecary, or other person, shall refill more than once prescriptions containing opium or morphine, or preparations of either in which the dose of opium shall exceed one-fourth grain or morphine one-twentieth grain, except with the verbal or written order of a physician." A violation of the law is declared a misdemeanor, punishable by a fine of not less than ten nor more than twenty-five dollars.

